# Anterior segment phenotypic changes in late-onset retinal degeneration

**DOI:** 10.1007/s00417-023-06041-0

**Published:** 2023-04-12

**Authors:** Leonardo Lando, Anne Xuan-Lan Nguyen, Randa Tsz Ha Li, Roly Megaw, Baljean Dhillon, Shyamanga Borooah

**Affiliations:** 1 Shiley Eye Institute, University of California San Diego, La Jolla, USA; 2 Department of Ophthalmology and Vision Sciences, University of Toronto, Toronto, Canada; 3 Faculty of Medicine, McGill University, Montreal, Canada; 4 Centre for Clinical Brain Sciences, School of Clinical Sciences, College of Medicine and Veterinary Medicine, University of Edinburgh, Edinburgh, Scotland, United Kingdom; 5 Princess Alexandra Eye Pavilion, NHS Lothian, Edinburgh, Scotland, United Kingdom; 6 MRC Human Genetics Unit, University of Edinburgh, Edinburgh, Scotland, United Kingdom

**Keywords:** Lens diseases, macular degeneration, CTRP5, genetic diseases

## Abstract

**Purpose:**

Late-onset retinal degeneration (L-ORD) is a rare retinal dystrophy with anterior segment (AS) abnormalities, including long anterior zonules (LAZ) and iris atrophy. This investigation aims to evaluate AS and longitudinal changes in a L-ORD cohort.

**Methods:**

Prospective, longitudinal study including L-ORD individuals (Ser163Arg) with ocular exam and standard slit-lamp photographs between 2011-2022. AS images were merged and assessed for LAZ number and zonule-free zone (ZFZ) radius. Other clinical findings were reported descriptively.

**Results:**

12 eyes (6 patients, 4 males), median aged 60.5 years (56-70) were included. Eyes had a median of 160 (11-372) LAZs, mainly localized superiorly (39%) and inferiorly (24%). There was a high inter-ocular correlation (r=0.98, p=0.001), no difference in LAZ count between eyes (p=0.34), and an inverse relationship between LAZ and age (r=-0.78; p<0.05). The ZFZ was a median of 2.1 mm (1.3-5.4), with no inter-ocular difference (p=0.34). ZFZ was correlated with age (r=0.85; p=0.03) but not with LAZ count per eye (r=-0.68; p=0.14). In a short-term follow-up of 5.9 years, a longitudinal analysis of 4 eyes showed a reduction in LAZ count to a median of 139.5 (67-169) (p=0.50) and a concomitant increase in the ZFZ measurement to a median of 2.2 (1.7-2.6) (p=0.17).

**Conclusion:**

This study confirms symmetric LAZs count and ZFZ in L-ORD, with ZFZ measurements smaller than in previous cohorts. A reduction in LAZs count and an increase in ZFZ with age were observed in two short-term follow-up patients. These findings require confirmation in cohorts with longer follow-ups.

## Introduction

Late-onset retinal degeneration (L-ORD) is a rare autosomal dominant macular dystrophy caused by mutations in the *C1QTNF5/CTRP5* gene. The gene is fully penetrant and is expressed in the eye at the level of the retinal pigment epithelium (RPE), lens epithelial cells, and ciliary body.^1–3^ Clinically, L-ORD is marked by nyctalopia manifesting around the 40s, retinal pseudodrusen, and delayed dark adaptation.^4–15^ In later stages, degeneration progresses with retinal and choroidal degeneration and macular atrophy, which leads to central vision loss.^2,4,6–16^ In some cases, the disease course may be complicated by choroidal neovascularization or cataract, which can further affect the visual status.^2,17,18^ During its natural history, L-ORD can mimic agerelated macular degeneration (AMD), whose prognosis and management is otherwise distinct.^11,13,15,18^

In addition to the posterior segment findings, L-ORD is known for anterior segment (AS) involvement.^2,6,7,14,19^ Some common AS abnormalities reported include zonules that atypically extend beyond the lens equator on the anterior side of the lens capsule, termed long anterior zonules (LAZs), and iris atrophic defects visualized by the transillumination on the slit lamp.^2,6,7,14,19^ These AS findings are important diagnostic clues for L-ORD as they may occur before fundus changes.^2^

AS findings in L-ORD remain only partially characterized, given the limited number of patients and lack of more extended longitudinal studies. In a recent series focusing on AS changes in L-ORD eyes, patients with the Pro188Thr variant exhibited predominant iris abnormalities compared to LAZs, which appeared distinct from previous descriptions in the Ser163Arg variant of L-ORD.^2,6,7,14,18,19^ This study aims to expand the understanding of the AS phenotype of L-ORD by analyzing AS changes in a cohort of LORD subjects.

## Methods

This prospective, longitudinal cohort study included patients with a molecular diagnosis of L-ORD (Ser163Arg) enrolled in a natural history study from 2011 to 2016 at the Princess Alexandra Eye Pavilion, Edinburgh, United Kingdom. Institutional research board approval was obtained from the research committee at Newcastle University (11/NE/0199). The study was conducted according to the tenets of the Declaration of Helsinki and the National Health Service Trust hospitals. Full informed consent was obtained from all participants.

As part of the clinical protocol, individuals underwent periodic ocular evaluation, which included a complete AS clinical exam with refraction (registered as spherical equivalent, SE) gonioscopy, pachymetry (Accutome, Lynwood, Washington, US), axial length (AL) measurement by A-scan (IOL Master 700, version 1.50, Carl Zeiss Meditec, Jena, Germany) and anterior segment imaging with a slit-lamp-mounted camera with flash (Canon EOS7D, Canon Inc, Tokyo, Japan). Cases were included if a clinical diagnosis of L-ORD was confirmed, molecular testing showed the patient carried the Ser163Arg variant, AS exam was completed, and two standard non-mydriatic AS pictures revealing LAZs and the peripupillary area were performed at baseline. For the purposes of this study, LAZ was defined as any zonule fiber extending beyond the pupillary border dynamically photographed, which entailed an anterior extension beyond the 2-mm landmark as classically proposed.^20^ In addition, AS images were required to have been acquired with retroillumination in dim light conditions, 16x magnification, and the flash reflex positioned at the central pupillary area around 3 and 6 o’clock meridians beyond the LAZs insertion, allowing for adequate LAZs visualization without blocking.

**Figure 1:**
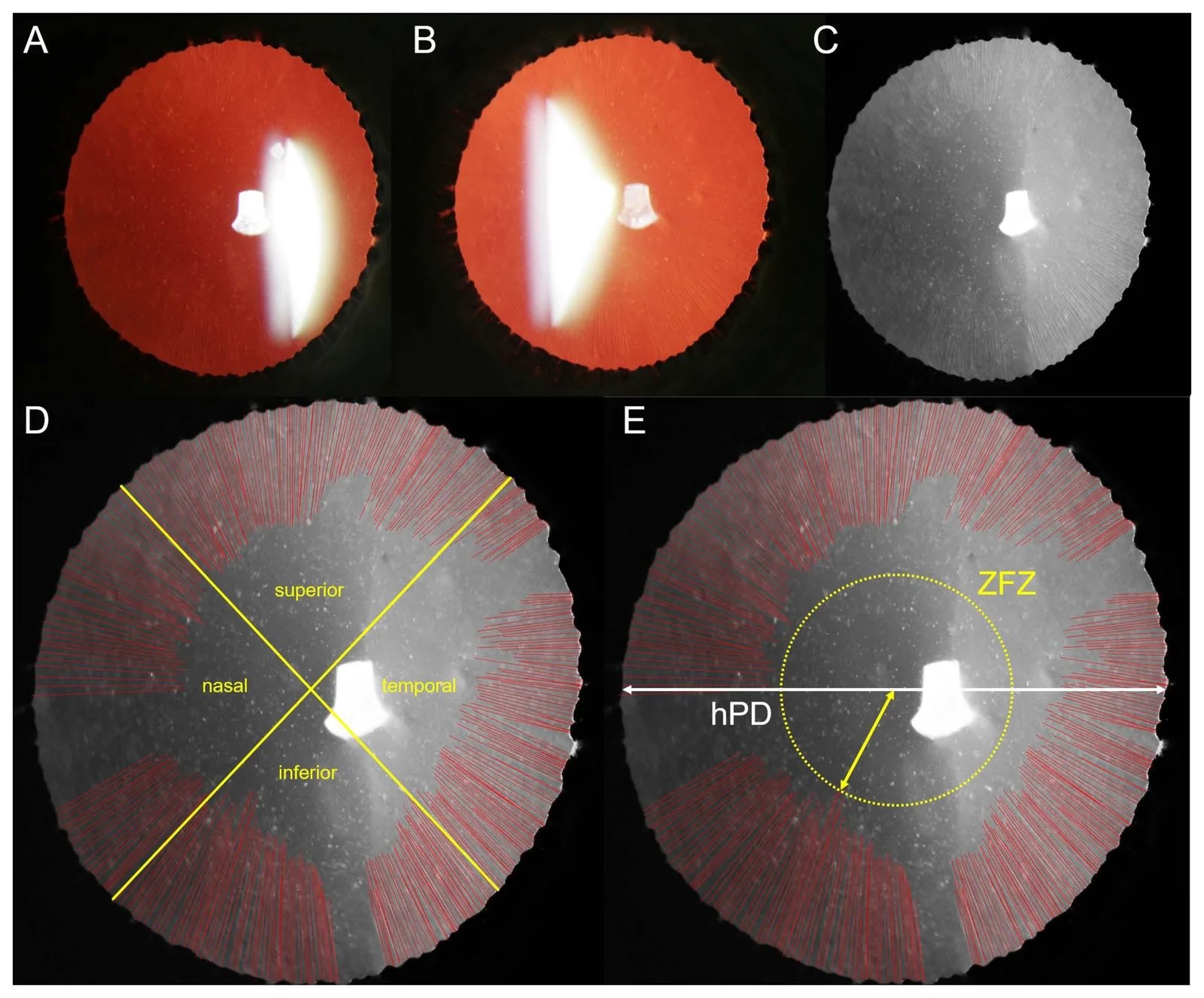
Method used for long anterior zonules (LAZ) counting and zonule-free zone (ZFZ) measurement. Two standard, non-mydriatic anterior segment photos obtained by transillumination were selected, one carrying the intrinsic flash artifact on the nasal half of the lens and another on the temporal half (A-B). Images were cropped, processed, and merged to outline the full LAZ extent without obstruction. The final image was converted to a gray scale for better LAZ visualization. As shown in this case, a final residual reflex was still noticeable without any repercussion to LAZs delineation (C). Subsequently, each zonular stria was marked by two independent graders (red lines), plotting points for LAZ counting. LAZ topography was determined according to four sectors delimited by two diagonal lines (yellow) geometrically traced, with the reference point consisting of the zonule touch to the pupillary margin (D). Finally, the ZFZ radius was measured using the centermost (longest) zonule distance to the lens center determined by the diagonal coordinates scaled to three average horizontal pupillary diameters (hPD) of 11.5-, 12.0-, and 12.5-mm (E).

After images were selected and checked for adequacy, image halves were processed and merged using an adapted methodology^21^ assisted by the Photomerge™ tool on Adobe Photoshop® (v.21.0.1, Adobe Systems) to overcome the flash artifact (Figure 1). Each merged image was carefully revised for consistency, referencing the original, nonmerged photos. Final merged pictures were converted into grayscale for better contrast in LAZ interpretation. Cases that had incomplete baseline medical records, inadequate AS capture, or resultant unsuitable photos after the merging process were excluded from the study.

For LAZ analysis, the final merged, gray-scaled images were square cropped (1:1) to the closest point adjacent to the pupillary borders and processed on MATLAB (R2021a). Diagonal coordinates were traced between image corners dividing four quadrants similar to previous investigations (superior/nasal/inferior/temporal).^19,21^ LAZs were then manually traced from the lens centermost point of insertion to the pupillary margin using a line tool by two independent readers (ophthalmologists). Based on the plotted points, the zonules were then counted and topographically located, using the striae termination at the pupillary margin (closest point to the equator) as the reference point. The LAZ pattern was also determined from final merged images and classified into segmental, nonsegmental or mixed, as previously published.^21,22^ The ZFZ radius was measured on the AS photos using the distance between the anterior end of the longest zonule to the central lens. Following a previous method, the values were scaled to three horizontal pupil diameters (hPD) of 11.5-, 12.0-, and 12.5-mm.^21^ For iris analyses, peripupillary defects were identified and classified descriptively following previous investigations.^6–8,14,19,23,24^

Demographic, clinical, and ocular parameters were identified. Correlations were calculated using Pearson chi-square using one LAZ or ZFZ measurement per patient (random choice). Comparisons between continuous variables were assessed by student’s t-test. In a small subset of eyes with follow-up AS photos, LAZ and ZFZ were similarly measured and compared using linear mixed modeling. Statistical calculations were performed on SPSS® (v.25; IBM, Chicago, IL, USA).

## Results

A total of 12 eyes from 6 patients were included in the baseline image analysis (Table 1). One case (2 eyes) was excluded due to inadequate baseline AS imaging. In the longitudinal analysis, only 4 eyes (2 patients) had available follow-up data and adequate AS imaging allowing for LAZ assessment. On baseline exam, subjects had a median age of 60.5 (56-70) years, comprising of 4 males and 2 females. To validate our methods, a test-retest (ICC=0.98, p<0.001) and inter-rater analysis (ICC=0.98; p< 0.001) for LAZ count was performed by two independent observers, confirming a high agreement. A similar analysis for the ZFZ area also revealed a high inter-rater agreement (ICC=0.97; p<0.001) and test-retest repeatability (ICC=0.95; p<0.001). Patients had a variable number of LAZs, with a median count of 160 (11-372) per eye. LAZs were located 360 degrees in the majority of individuals, being most prevalent in the superior (39%), inferior (24%), nasal (19%), and temporal (18%) quadrants. LAZ patterns were predominantly nonsegmental, and only one case displayed a mixed (segmental and nonsegmental) pattern. The ZFZ radius, scaled to three hPDs of 11.5-, 12.0-, and 12.5-mm, revealed medians of 2.0mm (1.2-5.2), 2.1mm (1.3-5.4), and 2.2mm (1.4-5.6), respectively. The median AL was 23.0 (19.6-25.8).

**Figure 2:**
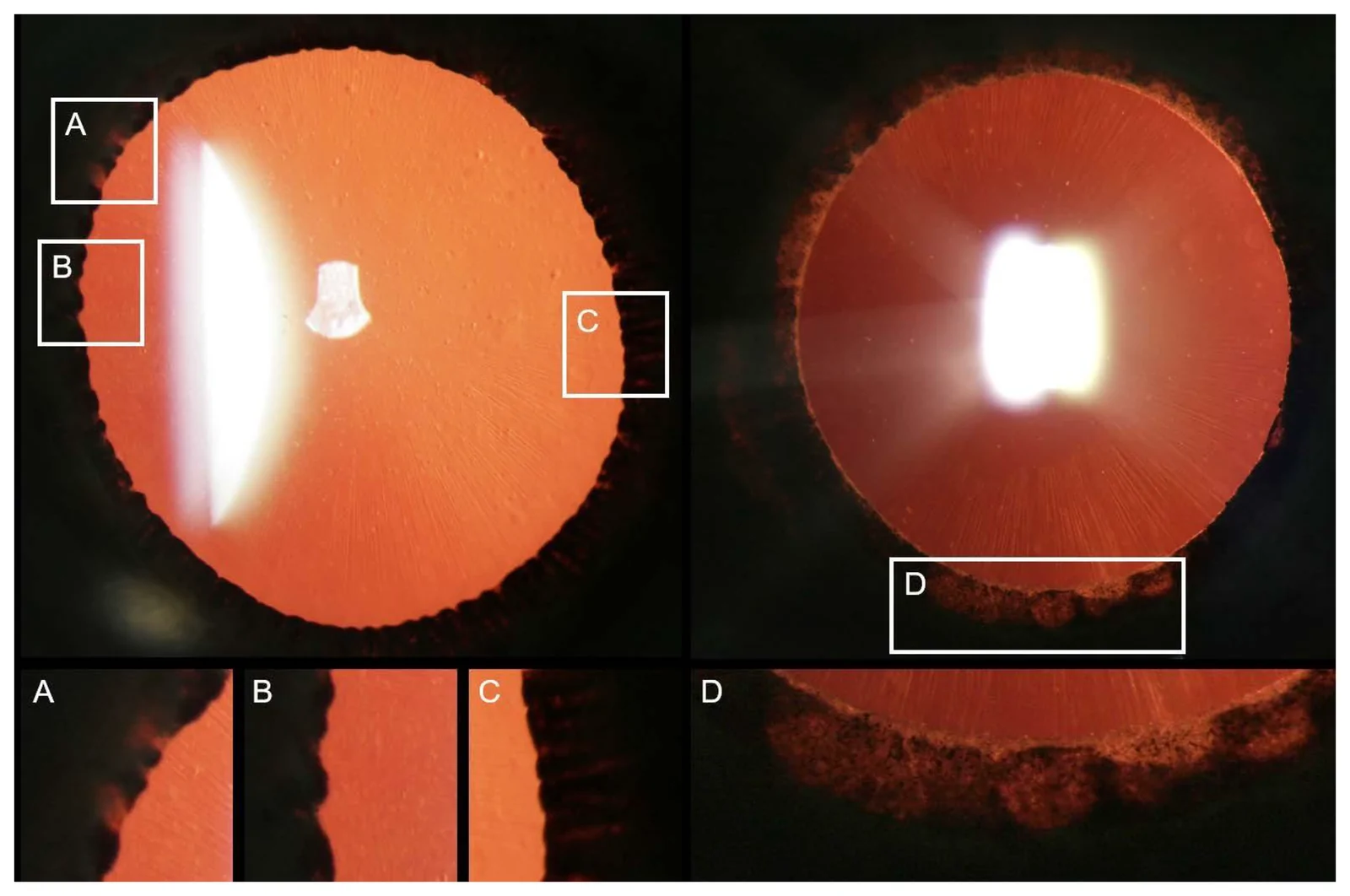
Photos of two different patients illustrating the four different types of iris atrophic defects, which were variably present in eyes with late-onset retinal degeneration and were globally categorized into pupillary ruff rarefaction (A), notched defects (B), radial streaks (C), and patchy atrophy (D).

Iris color was measured using a standard panel.^25^ Irises were classified mainly within the blue spectrum with slight variations, as detailed in Table 1. SE also varied amongst cases, with a median at low myopia [-0.25, (-5.37)-(+1.75)]. Iris transillumination defects were identified in the peripupillary margin in 11/12 eyes and mainly consisted of a mixed type of involvement in the form of pupillary ruff rarefaction (11/11), notched defects (6/11), patchy atrophy (6/11), and radial streaks (2/11) (Figure 2). All cases displayed deep anterior chambers (grade 4) by van Herick grading. On gonioscopy, the angle was open in all cases, with pigmentation clinically graded as mild (4/12), moderate (4/12), and marked (4/12). Corneal thickness by pachymetry showed a median value of 536 (496-594) µm. No evidence suggesting pigment dispersion on the endothelium was noticed in any studied eyes.

In the exploratory analysis, there was a strong correlation in LAZ number between right and left eyes (r=0.98, p=0.001), with no statistically significant difference between eyes (p=0.34). Additionally, no statistically significant difference was found between the ZFZ radius comparing same-patient eyes (p=0.34). Altogether, this indicated that LAZs were symmetric. LAZ counts showed a strong correlation with pachymetry values (r=0.92; p=0.01) and had an inverse relationship with age (r=-0.78; p<0.05). ZFZ was positively associated with age (r=0.85; p=0.03) but not with total LAZ count (r=-0.68; p=0.14). No statistically significant correlation was obtained between LAZ and SE (r=0.01; p=0.77), LAZ and AL (r=0.21; p=0.55), ZFZ and SE (r=0.36; p=0.25), or ZFZ and AL (r=-0.64; p=0.05).

**Figure 3:**
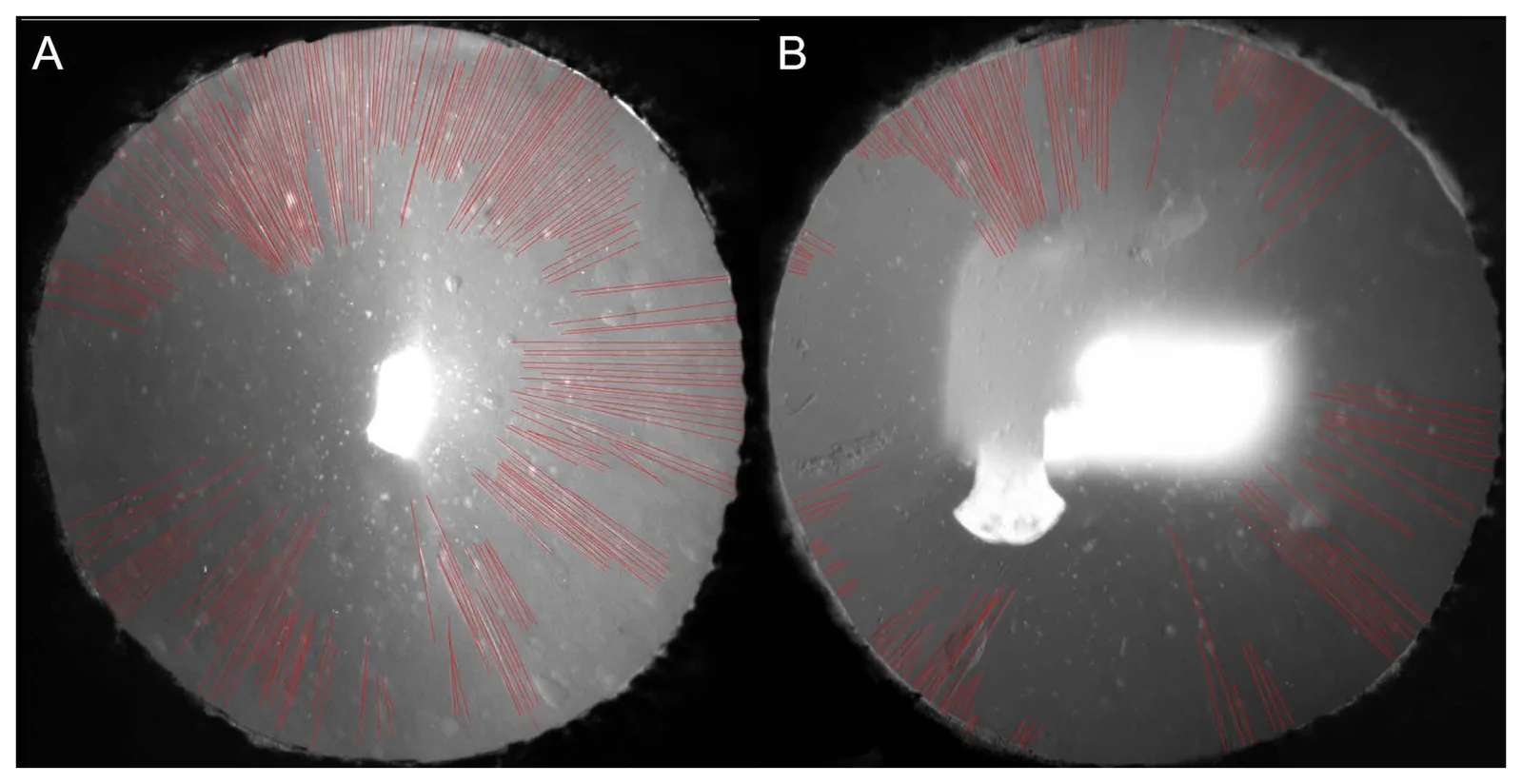
Comparative follow-up images of a 56-year-old female (case 5) left eye with long anterior zonules exemplify the progressive decline in the zonules count from baseline (A) to 5.9 years later (B). During this time, a simultaneous increase in the zonule-free zone was seen, likely consequential to a primary loss of the longer zonular striations. A global reduction in the length of other zonules over 360 degrees appeared to be part of this process of LAZ remodeling with time.

A longitudinal analysis was carried out on the 4 eyes (2 patients) available from a visit after 5.9 years. In these individuals, LAZ count showed a reduction in number from a baseline median of 160 (74 and 240) to 139.5 (67 and 169) at follow-up (p=0.50). Additionally, there was an increase in the ZFZ (scaled to 12 mm) from median 1.6 (1.31.9) to 2.2 (1.7-2.6) (p=0.17) (Figure 3). LAZ decrease evolved diffusely in all quadrants in the 4 eyes studied.

## Discussion

In keeping with previous clinical investigations, this study confirms that AS abnormalities are commonly seen in L-ORD, as demonstrated by all eyes included in the study. In addition, the analyses confirm a high level of symmetry of LAZ and ZFZ metrics between eyes, supporting previous clinical, image-based, and histopathological studies that the disease is symmetrical in nature.^2,4–16,18^ Longitudinally, LAZs count appears to manifest a progressive decrease, though patient numbers were small for this conclusion. The loss of the more medial LAZ seems to have resulted in an increase in the ZFZ.

Ayyagari et al^16^ were among the first to identify that L-ORD patients manifested AS changes in addition to retinal degeneration while studying family members with L-ORD. In 6 out of 10 patients, zonular-like striations on the anterior lens capsule were noted by the authors, who realized that in 3 of these patients, no evidence of fundus abnormalities was present at the time of the diagnosis of the zonular findings. The identification of the *C1QTNF5/CTRP5* mutation in 2003, followed by an understanding of the expression of C1QTNF5 protein within the eye, later assisted in explaining the AS changes.^1,3^ In 2005, Subrayan et al^6^ used the expression ‘long anterior zonules’ to describe the zonule features in L-ORD while describing 3 subjects of the same family with clinically evident LAZ and iris transillumination defects.

The mechanisms of LAZ development in L-ORD are incompletely understood. Zonular fibers are thought to form around the third gestational month, consisting of approximately 10-12-nm microfibrils mainly composed of polymerized fibrillin, which participates in the physiology of lens accommodation.^26^ Some authors have postulated that the atypical zonules could represent remnants of a pupillary membrane, which has previously been associated with LAZ,^27,28^ although other cohorts of children with persistent pupillary membrane did not indicate this relationship.^29^ Similarly, the presence of a pupillary membrane has not been observed as part of L-ORD LAZ phentoype^2,6,7,14^ and, as such, the exact timepoint of LAZ formation remains unknown.

Animal models and clinical observations suggest that the abnormal zonular complex in L-ORD individuals might be a later event in life rather than a developmental malformation or embryonic remnant.^3,18^ This is supported by the fact that the first LAZ cases described in non-L-ORD eyes were found in patients in their early adulthood, around their 20s, most of them without any other significant ocular findings.^28^ In L-ORD, one of the youngest cases of LAZ was reported at the age of 24 to an individual known to be part of a family with molecularly confirmed L-ORD in which a total of 18/55 members had LAZ.^2^ Importantly, in L-ORD patients diagnosed with Pro188Thr variant, LAZ was not a significant phenotypic marker, being identified in only 2 of the 26 examined cases.^14^ This might suggest that the S163R variant may have a greater preponderance for generating LAZ.

Physiologically, the zonular insertions occur at 1.5-2 mm anterior to the lens equator, whereby a dense layer of longitudinally-disposed fibers is created on the capsule surface.^20,26,30^ In the dynamic exam on the slit lamp, usually facilitated by retroillumination, these fibers tend to remain covered by the iris stroma, becoming visible only under pharmacologic mydriasis or in situations of lens subluxation such as in trauma. In situations where zonule fibers extend beyond the 2-mm landmark, the LAZs are found present.^20^ In the general population, the LAZ trait has been of particular interest as initial reports hypothesized a risk of surgical complications during capsulorhexis for cataract extraction.^31^ However, a previous study suggests that the LAZ does not play a structural role in stabilizing the lens capsule in the adult, as a number of cataract surgeries have been performed without apparent adverse effects on post-operative lens stability.^17^

Although typical in L-ORD, LAZs are not pathognomonic of this condition, being encountered as an incidental normal finding in approximately 2% of healthy eyes in different ethnic groups.^20–22,30,32–34^ In different studies, LAZs were associated with age above 50 years, female sex, hyperopia, short axial length, and several ocular conditions, including increased intraocular pressure, pigment dispersion syndrome, plateau iris configuration, open- and narrow-angle glaucoma.^20–22,30,32–35^ In L-ORD, none of these conditions have been consistently identified, though one early L-ORD study^2^ raised concerns for glaucoma development in individuals with LAZ. In subsequent more extensive familial and clinical studies, glaucoma did not appear to be a reportable condition amongst L-ORD patients, despite limitations from the small number of cases and study methods.^6,7,9–11,13,14^ Because of the close physical relationship of LAZs and the iris promoting pigmentation of the zonules, the descriptor ‘pigmented lens striae’ has been occasionally employed in L-ORD^2^ as well as in other LAZ populations^26,31^.

In this study, LAZ count was shown to vary in L-ORD eyes, with a median of 160 zonules per eye. Using a similar methodology, Roberts et al^21^ estimated a median of approximately 50 zonules per eye in 59 individuals manifesting the LAZ trait at a mean age of 70 (53-91) years, the vast majority being women. In the same study, the authors calculated a mean ZFZ measurement of approximately 3 mm, contrasting with the 2 mm measurement in this cohort of L-ORD cases.^21^ Topographically, zonules were predominantly located in the superior quadrant both in individuals with LAZ trait and, as demonstrated, in L-ORD, a finding that lacks explanation and requires further investigation.^19,21^ In our analysis, no relationship was encountered between LAZ count and ZFZ measurements in L-ORD eyes, similar to the findings from other study groups looking at LAZs.^19,21^ It is known that zonules decrease with age in eyes devoid of LAZs.^26^ The negative correlation between LAZ count and age was interesting and possibly indicated a novel finding that these patients may experience a progressive loss of zonules over time.^19,21^

Beyond LAZs, this study also focused on evaluating the iris atrophic defects, which were also reported by previous authors.^6–8,14,19^ In our cohort, iris changes were noted at different levels of severity and could be classified into 4 major clinical types: pupillary ruff rarefaction, patchy atrophy, notched defects, and radial streaks. These patterns, which were present in a mixed way in most eyes, still require further clarification regarding their degree of severity and functional impairment. In all 6 cases, iris changes occurred simultaneous to LAZ, despite no overt relationship between the areas of LAZ and iris atrophy that suggested a pigmentary dispersion mechanism.

Particularly, iris abnormalities have been well characterized as part of the Pro188Thr variant.^14^ In a cohort with 23 L-ORD individuals carrying this variant, De Zaeytijd et al^14^ reported on abnormal iris features in at least 9 cases, which mostly carried midperipheral patchy iris defects and/or moth-eaten appearance of the pupillary sphincter with concomitant poor dilation. The authors also identified two patients with poor dilation regardless of any visible iris change. Unfortunately, the dilation function was not evaluated as part of the cases presented in our series.

The main limitations of this study include the inherent and uncontrolled variations in image acquisition using retroillumination, resulting in a potential reduction or obscuration of data by the photography merging technique. In addition, the small patient sample and the limited number of cases with consistent follow-up data meant that conclusions would need further clarification. These studies are currently ongoing with a larger number of patients for comparison.

In summary, this study quantifies LAZ parameters and descriptively explores iris defects in L-ORD. This series confirms symmetry of LAZ in L-ORD and suggests longitudinal loss of LAZ. The present study also suggests that iris involvement is common in this cohort of L-ORD patients.

## Figures and Tables

**Table 1:** Baseline demographics and clinical details of the anterior segment features of 6 patients with late-onset retinal degeneration.

Case	Age	Sex	Ethnicity	Refraction (SE)	LAZ counting	Quadrants with LAZ	LAZ pattern	ZFZ (scaled for 12 mm pupil)	Iris color (same in both eyes)	Peripupillary iris defects	Type of peripupillary defect ^A^	Axial length (mm)	Pachymetry (pm)	Angle depth (Van Herick)	Degree of angle pigmentation (1-4)
1	67	M	Caucasian	OD: +0.25 OS: +0.75	OD: 110 OS: 93	OD: S, I, N, T OS: S, I, N, T	OD: nonsegmental OS: nonsegmental	OD: 3.14 OS: 2.80	Blue with peripupillary brown	OD: Yes OS: Yes	OD: 1,3 OS: 1,3	OD: 24.3 OS: 24.4	OD: 531 OS: 539	OD: 4 OS: 4	OD: 2 OS: 2
2	59	M	Caucasian	OD: -2.00 OS: -2.25	OD: 372 OS: 365	OD: S, I, N, T OS: S, I, N, T	OD: nonsegmental OS: nonsegmental	OD: 2.53 OS: 2.23	Brown with peripheral green	OD: Yes OS: Yes	OD: 1,3,4 OS: 1,3,4	OD: - OS: -	OD: 594 OS: 590	OD: 4 OS: 4	OD: 4 OS: 4
3	56	F	Caucasian	OD: +1.75 OS: +1.50	OD: 245 OS: 235	OD: S, I, N, T OS: S, I, N, T	OD: mixed OS: mixed	OD: 1.92 OS: 1.89	Blue with peripupillary brown	OD: Yes OS: Yes	OD: 1,2 OS: 1,2	OD: 23.1 OS: 23.0	OD: 569 OS: 556	OD: 4 OS: 4	OD: 1 OS: 1
4	62	M	Caucasian	OD: -5.37 OS: -4.50	OD: 74 OS: 112	OD: S, I, N, T OS: S, I, N, T	OD: nonsegmental OS: nonsegmental	OD: 1.74 OS: 1.48	Light blue	OD: Yes OS: Yes	OD: 1,3 OS: 1,2	OD: 25.8 OS: 25.4	OD: 532 OS: 532	OD: 4 OS: 4	OD: 1 OS: 1
5	56	F	Caucasian	OD: -0.25 OS: -0.25	OD: 240 OS: 208	OD: S, I, N, T OS: S, I, N, T	OD: nonsegmental OS: nonsegmental	OD: 1.90 OS: 1.30	Blue with peripupillary brown	OD: No OS: Yes	OD: n/a OS: 1,2,3	OD: 22.6 OS: 22.6	OD: 556 OS: 533	OD: 4 OS: 4	OD: 3 OS: 3
6^B^	70	M	Caucasian	OD: -0.25 OS: -0.75	OD: 36 OS: 11	OD: S, T OS: S, N	OD: nonsegmental OS: nonsegmental	OD: 5.38 OS: 3.02	Light blue	OD: Yes OS: Yes	OD: 1,2 OS: 1,2	OD: 19.6 OS: 19.8	OD: 506 OS: 496	OD: 4 OS: 4	OD: 4 OS: 4

SE: spherical equivalent; LAZ: long anterior zonules; OD: right eye; OS: left eye; ZFZ: zonule-free zone

A 1: Pupillary ruff rarefaction; 2: Patchy atrophy; 3: Notched defects; 4: Radial streaks

B Primary-open angle glaucoma diagnosis

## Value Statement

### What was known

- Patients with late-onset retinal degeneration (L-ORD) manifest anterior segment changes.
- While previous studies have explored these changes descriptively, no investigation has quantified LAZ parameters and explored specifics of iris transillumination defects.
- No previous investigation, to our knowledge, has either described or quantified longitudinal changes in LAZs as part of L-ORD.

### What this paper adds

- Iris abnormalities and long anterior zonules are part of the L-ORD phenotype in individuals with the Ser163Arg variant.
- Four types of iris transillumination defects were identified amongst the 12 studied eyes, which further revealed longer LAZs compared to other populations with the LAZ trait
- A follow-up revealed a reduction in LAZ count with possible primary loss of longer zonules, as revealed by an increase in the central zonule-free zone.

## Abbreviations

AL: axial length
AMD: age-related macular degeneration
AS: anterior segment
hPD: horizontal pupil diameter
LAZ: long anterior zonules
L-ORD: late-onset retinal degeneration
RPE: retinal pigment epithelium
SE: spherical equivalent
ZFZ: zonule-free zone
